# A unified 3D map of microscopic architecture and MRI of the human brain

**DOI:** 10.1126/sciadv.abj7892

**Published:** 2022-04-27

**Authors:** Anneke Alkemade, Pierre-Louis Bazin, Rawien Balesar, Kerrin Pine, Evgeniya Kirilina, Harald E. Möller, Robert Trampel, Johan M. Kros, Max C. Keuken, Ronald L. A. W. Bleys, Dick F. Swaab, Andreas Herrler, Nikolaus Weiskopf, Birte U. Forstmann

**Affiliations:** 1 Integrative Model-Based Neuroscience Research Unit, University of Amsterdam, Amsterdam, Netherlands.; 2 Department of Neurophysics, Max Planck Institute for Human Cognitive and Brain Sciences, Leipzig, Germany.; 3 Department of Neurology, Max Planck Institute for Human Cognitive and Brain Sciences, Leipzig, Germany.; 4 Department of Neuropsychiatric disorders, Netherlands Institute for Neuroscience, an Institute of the Royal Netherlands Academy of Arts and Sciences, Amsterdam, Netherlands.; 5 Neurocomputation and Neuroimaging Unit, Department of Psychology and Educational Science, Free University Berlin, Habelschwerdter Allee 45, Berlin 14195, Germany.; 6 NMR Methods Development Group, Max Planck Institute for Human Cognitive and Brain Sciences, Leipzig, Germany.; 7 Department of Pathology, Erasmus Medical Center, Rotterdam, Netherlands.; 8 Department of Anatomy, University Medical Center Utrecht, Utrecht University, Utrecht, Netherlands.; 9 Department of Anatomy and Embryology, Maastricht University, Maastricht, Netherlands.; 10 Felix Bloch Institute for Solid State Physics, Faculty of Physics and Earth Sciences, Leipzig University, Linnéstraße 5, Leipzig 04103, Germany.; 11 Wellcome Centre for Human Neuroimaging, Institute of Neurology, University College London, 12 Queen Square, London WC1N 3AR, UK.

## Abstract

We present the first three-dimensional (3D) concordance maps of cyto- and fiber architecture of the human brain, combining histology, immunohistochemistry, and 7-T quantitative magnetic resonance imaging (MRI), in two individual specimens. These 3D maps each integrate data from approximately 800 microscopy sections per brain, showing neuronal and glial cell bodies, nerve fibers, and interneuronal populations, as well as ultrahigh-field quantitative MRI, all coaligned at the 200-μm scale to the stacked blockface images obtained during sectioning. These unprecedented 3D multimodal datasets are shared without any restrictions and provide a unique resource for the joint study of cell and fiber architecture of the brain, detailed anatomical atlasing, or modeling of the microscopic underpinnings of MRI contrasts.

## Teaser

A 200-μm scale 3D reconstruction of the human brain was created based on ultrahigh-field quantitative MRI and light microscopy.

## INTRODUCTION

An exhaustive map of the human brain has been a long-sought goal of neuroanatomists. From the outset, the need to create systematic descriptions of the human brain has been clear. Entire research groups dedicated themselves to these endeavors, which led to the development of neuroanatomical methodology (e.g., immunohistochemistry) and the creation of classical maps by Brodmann (*1*), Vogt and Vogt (*2*), and von Economo and Koskinas [(*3*), for a review, see (*4*)]. Today, these anatomical mapping approaches can be combined with modern brain imaging and computing techniques. These efforts range from inserting the classic microscopy preparations into existing modern anatomical templates (*5*, *6*) to the creation of new three-dimensional (3D) atlases of increasingly smaller structures from submillimeter resolution in vivo or postmortem magnetic resonance imaging (MRI) [e.g., (*7*–*11*)]. However, it remains a scientific tour de force to combine the exquisite detailed microscopy images and reconstruct them into an integrated view of the 3D anatomy of the human brain (*12*). Until now, only one 3D whole-brain map derived from microscopy with whole-brain coverage is openly available, providing details of cytoarchitecture from a single type of histological staining at the impressive resolution of 20 μm across sections and 21 μm in-plane (*13*). Other recent attempts have managed to provide fully annotated stained sections from three different microscopy contrasts and mapped the annotations to the montreal neurological institute (MNI) template (*14*, *15*), or aligned microscopy and 3-T MRI covering the entire brain at 400 μm (*16*).

## RESULTS AND DISCUSSION

We present the first openly accessible 3D whole-brain map of multiple microscopy contrasts and 7-T quantitative multiparameter MRI reconstructed at 200 μm (movie S1). We have included all available sections for staining, such as the BigBrain reconstruction (*12*, *13*). The integrated series of images depicts nerve fibers, neuronal and glial cell bodies, as well as immunolabeling of interneuron populations, coregistered into a common space at 200-μm resolution. For each individual staining procedure, we provide a 600- to 1000-μm sampling interval, which provides sufficient detail for the comparison of mRNA and immunoreactivity in prominent hypothalamic nuclei (*17*–*19*). For the blockface, all sections were sampled. We would like to note that for various purposes, such as the identification of small nuclei or the mapping of the vasculature, data from multiple staining procedures can be collapsed, thereby providing full coverage of the brain. MRI acquired before sectioning consists of multiple quantitative MRI parameter maps with an isotropic resolution of 400 μm. All coregistered maps are freely accessible, offering the opportunity to explore the anatomical richness of the human brain from complementary perspectives. Our efforts provide a direct comparison between histological markers through whole-brain coverage and quantitative ultra-high field (UHF) MRI results. Our work provides a major step forward and extends previous efforts, such as those published by Ding *et al.* (*14*).

**Fig. 1.**
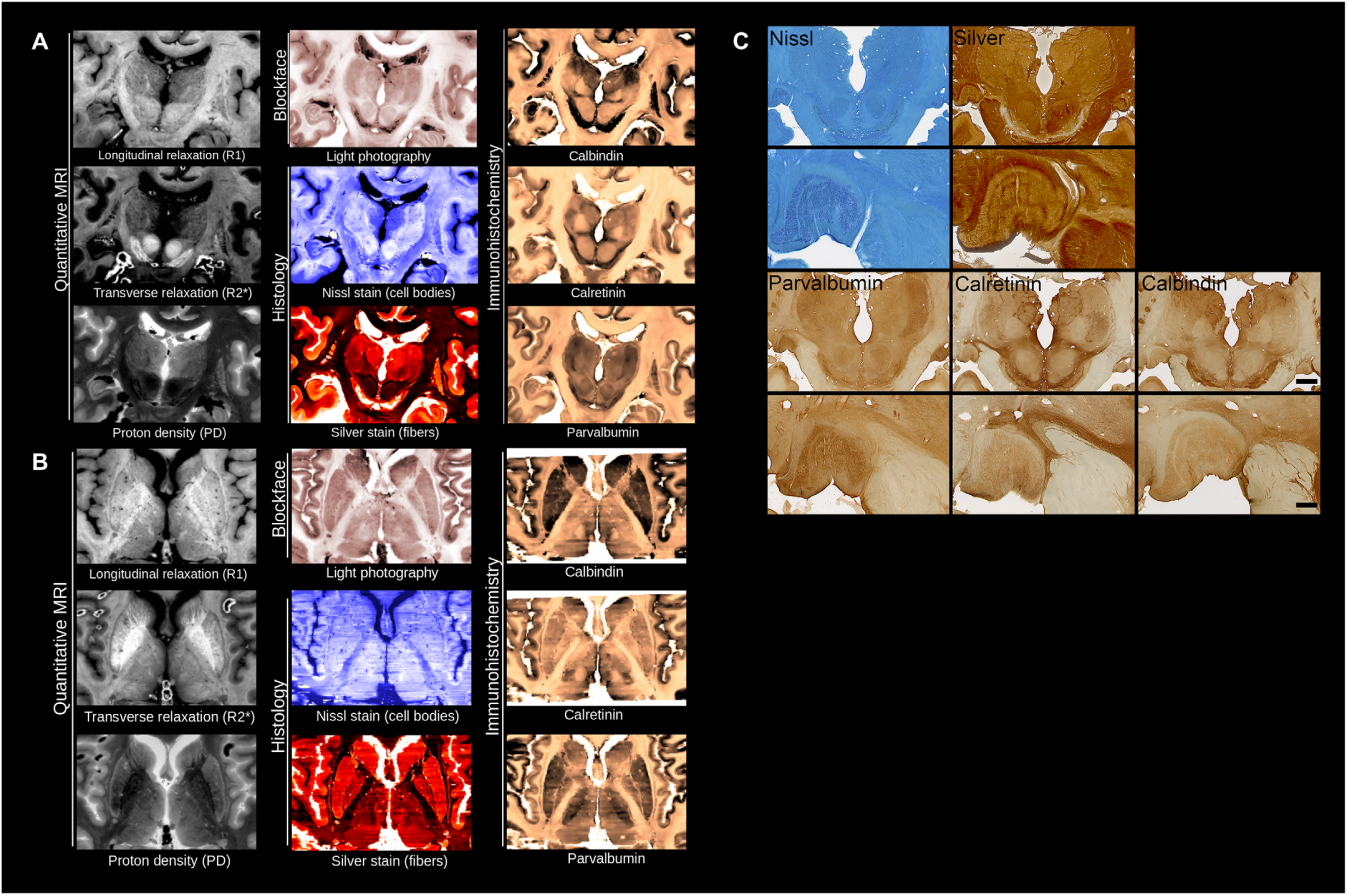
Anatomical detail obtained from reconstructed human brains. (**A** and **B**) Coronal (A) and axial view (B) for the three MRI contrasts, blockface images, and five microscopy stains. (**C**) Illustrations of single sections for Nissl and silver (Bielschowsky) staining, and parvalbumin, calretinin, and calbindin immunoreactivity in the thalamus (scale bar, 1 cm). High-power magnifications (bottom) show high-power magnifications of the lateral geniculate nucleus of the thalamus. Scale bar, 250 μm.

The inclusion of five microscopy labels, blockface images, and three quantitative MRI contrasts provides a wealth of anatomical information (Fig. 1). The full-brain coverage allows for detailed and comparative analyses of architectonic features for mapping the cortical laminar structure (*20*–*23*). A second important application is the atlasing of small brain structures that cannot be discerned on MR images because of their small size or limited MRI contrast (*24*). The inclusion of the smaller structures including (hypo-)thalamic nuclei and deep cerebellar nuclei in MRI atlases will provide new insights into these understudied or poorly defined structures (*24*–*26*). The availability of coaligned multiple microscopy contrasts will improve the localization and differentiation of these structures in MRI. Such detailed anatomical references are also of importance to surgical planning for deep brain stimulation (*27*, *28*).

**Fig. 2.**
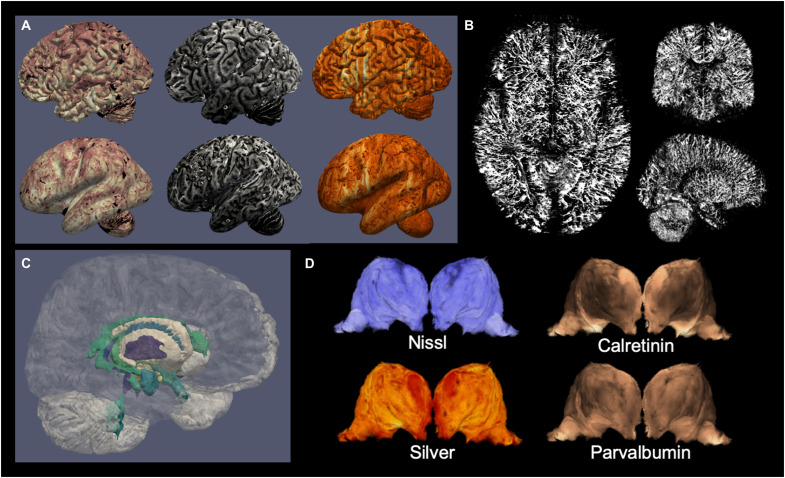
Examples of information derived from the dataset of specimen no. 15-2017. (**A**) Cortical maps from the dataset: blockface (left), quantitative R2* (middle), and parvalbumin immunohistochemistry (right), sampled at the midcortical surface in fully folded (top) or inflated (bottom) views; (**B**) reconstructed blood vessels extracted from the coregistered stainings; (**C**) automated cortical and subcortical parcellations; (**D**) different stainings outline different thalamic nuclei boundaries.

This dataset provides an essential link for studies on the relationship between the myelo- and cytoarchitecture of the human brain, which can now be compared to quantitative MRI parameters through the coregistration across MRI and microscopy modalities. Comparing the cyto- and myeloarchitecture of the human brain has been a long-standing goal of neuroanatomy (*29*), and this dataset provides cell and fiber information over the entire brain in the same specimen. Interest in myeloarchitecture has been renewed by its relationship to MRI contrasts (*30*–*33*) and its involvement in mechanisms of brain plasticity (*34*, *35*). Using processing techniques adapted from MRI cortical analysis tools, MRI and microscopy contrasts can already be mapped on cortical surfaces (Fig. 2A). The combination of acquired 7-T MRI and microscopy data can facilitate building biophysical models linking microarchitecture and postmortem MRI to eventually extract microarchitectural information directly from in vivo MRI datasets (*36*). Understanding the microscopic detail underlying MRI, such as the anatomical detail provided for the lateral geniculate nucleus (Fig. 1), can, for instance, shed light on differences between structures in terms of variance observed in quantitative MRI parameters within small inhomogeneous brain structures (*26*). Some of the architecture of the brain remains largely unexplored, such as the angio-architecture (*37*–*39*). By detecting vessels in the multiple included stainings, we would be able to reconstruct whole-brain vascular maps (Fig. 2B) or use the data for automated delineations of subcortical structures (Fig. 2C). In structures such as the thalamus that have already been parcellated on the basis of various in vivo MRI contrasts, there can be discrepancies due to the fact that some borders show limited visibility with different approaches (*25*). With multiple immunohistochemical contrasts, anatomical boundaries become unequivocal and can be further compared to MRI contrast variations (Fig. 2D). The careful delineation of individual thalamic structures on the microscopy data and the subsequent transfer of the created masks onto the MRI data could inform new studies on the local differences in quantitative MRI parameters, which could be used to feed into algorithms trained to identify thalamic nuclei in individual MRI contrasts. In addition, the level of detail achieved by microscopy allows validation of MR initiatives for the imaging of U-fibers (*40*, *41*). A recent finding of increased iron concentration in the superficial white matter was linked to U-fibers (*42*), but the spatial organization of this relationship has not yet been mapped. The combination of the Bielschowsky stain with quantitative R2* MRI will allow to build such a map and study its relationship with cyto- and myeloarchitecture of the cortex. Last, the information contained in such anatomical datasets is invaluable for building large-scale computational models of the brain and study emergent behavior (*43*, *44*).

**Fig. 3.**
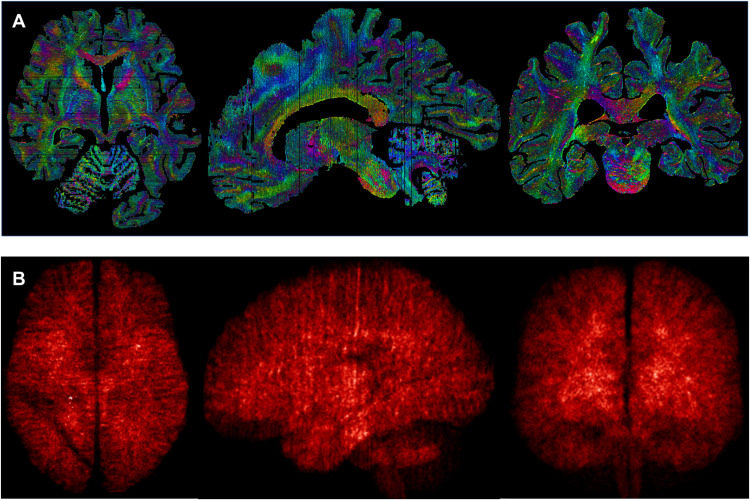
Example of applications using the full range of available resolutions. (**A**) Shape tensor analysis of the different sections at the 20-μm scale, recombined to the 200-μm space of the blockface image; (**B**) density maps of blood vessels detected from the background of the 20-μm sections realigned in 3D at 200 μm, projected, and averaged into MNI space at 0.5 mm.

Thanks to the high-quality 3D reconstruction, the data can be coregistered to MR images and further processed with MRI analysis toolboxes. As an example, we provide a version of the maps coregistered to MNI2009b (*45*), a segmentation of the cerebral and cerebellar cortices with the automated multiple object geometric deformable model (MGDM) and cortical reconstruction using implicit surface evolution (CRUISE) methods (*46*, *47*), and an automated parcellation of 17 subcortical structures with multi-contrast anatomical subcortical structures parcellation (MASSP) [(*48*), see Table 1]. The provided coregistration to MNI2009b space enables researchers to transfer publicly available brain maps and atlases to the microscopy sections. The parcellations of brain structures we provide here have been obtained by adapting and optimizing software tools originally developed to handle in vivo MRI data. As a result, these constitute a starting point for more elaborate whole-brain analyses but should be inspected critically for specific applications. We would like to note that manual delineation remains the gold standard for detailed neuroanatomical studies. Furthermore, the multiple scales of the dataset can be combined to provide whole-brain maps of microstructural features. Here, we present preliminary mappings of the mesoscopic vasculature detected on the 20-μm microscopy sections, reassembled in 3D in the 200-μm resolution blockface space, and coregistered to MNI space as well as maps of local shape tensor directionality for estimation of fine details of axonal architecture (*49*, *50*) estimated from the sections and recombined into blockface space (Fig. 3).

**Table 1.** Description of publicly available data and software.

**Data**	**Description**	**DOI**
Original stains	Archive of .tiff files of the microscopy stains in full resolution and color, including transformation maps to 3D blockface space	10.21942/uva.16844500
		10.21942/uva.16843039
3D reconstructions	3D stacked images of the stains and MRI in ~200-μm- resolution space of the blockface image	10.21942/uva.16834114
		10.21942/uva.14260064
MNI coregistrations	Stains, blockface, and MRI data in MNI2009B space at 0.5-mm resolution	10.21942/uva.16834186
		10.21942/uva.14260088
Automated parcellations	Parcellation results from the whole brain, cortical surface, and subcortical labeling algorithms	10.21942/uva.16834159
		10.21942/uva.14260079
Registration scripts	Python scripts for the various registration, mapping, and parcellation techniques used	10.21942/uva.16834132
		10.21942/uva.14260673

This unprecedented 3D multimodal dataset is shared without any restrictions and provides a unique resource for the joint study of cell and fiber architecture of the brain, detailed anatomical atlasing, or modeling of the microscopic underpinnings of MRI contrasts. The 3D multimodal dataset offers a unique view on the human brain. Cell and fiber architecture of the brain and ultrahigh-field quantitative MRI of two human specimens are reconstructed in an individual anatomical space. Previous studies either apply spatial interpolation to compensate for incomplete coverage or provide high sampling coverage for smaller tissue blocks only (*51*–*54*). In addition, work by others (*16*, *55*, *56*) provides a pipeline that has achieved whole-brain coverage in >400-μm-thick sections and combines microscopy with 3-T MRI. The anatomical expertise and equipment necessary to perform reliable cutting and staining of large microscopy section is scarce, and coregistration of such high-resolution sections presents substantial computational challenges (*12*). This dataset does not include microscopy at the cellular level, yet the order of magnitude and richness of contrasts provided here can bridge between microscopy in thin sections cut from small tissue blocks and the context of the whole brain. Postmortem research is inherently limited by low numbers of observations, which precludes the creation of probabilistic atlases, and is prone to potential bias due to antemortem illness, pharmacological treatment, cause of death, as well as postmortem interval, fixation procedures (including the introduction of air bubbles), and the storage time. A case report comparing antemortem MRI with MRI on in situ, unfixed tissue showed that whole-brain effects can occur on volumetric measurements, cortical thickness, and diffusion measures. Potentially, part of these differences were caused by the process of dying (*57*).

Although we cannot make any final claims based on two brains, we can provide a comparison between the two specimens. Our datasets combine at 200 μm a manageable and comprehensive 3D brain volume totaling five microscopy stainings, with in-plane levels of 21 μm detail in the microscopy sections, and three quantitative MRI contrasts. The 0.2-mm data can be inspected in 3D neuroimaging viewers, such as medical image processing, analysis and visualization (MIPAV), a general-purpose medical imaging visualization suite (*58*), which can handle the large size of the images. In addition to the processed images, we share the 1200 dots per inch (dpi) raw .tiff images, together with the corresponding transforms to 3D blockface space. Our efforts provide a unique direct comparison between histological markers and quantitative UHF MRI results, which represents a major step forward and extending previous research. With this combination of (immuno-)histochemical and MRI contrasts, the data can provide a common frame of reference to bring together studies focusing on a single anatomical structure in smaller tissue samples. We aim to offer researchers worldwide a foundational resource to bridge microanatomy, neuroimaging, and systems neuroscience.

## MATERIALS AND METHODS

Two whole human head specimens (female, nondemented, 75 years old, no. 15-2017 and female, 59 years old, no. 12-2017; postmortem interval <24 hours) were obtained from the body donation program of the University of Maastricht following a whole-body perfusion, for which written consent was obtained during life, and in accordance with the Dutch Burial and Cremation Act. Quantitative MRI maps of relaxation rates (R1, R2*) and proton density (Fig. 1) (*59*, *60*) were acquired with 400-μm isotropic resolution at 7 T, similarly to ultrahigh in vivo multimodal quantitative MRI acquisitions (*42*). After MRI scanning autopsy was performed and following sucrose protection, the brain was frozen on dry ice in Tissue-Tek. Blockface images were acquired for all sections during 200 μm serial coronal cutting with a resolution of 500 pixels per inch. Blockface images were acquired with a camera mounted perpendicular to the tissue block, thereby reducing barrel and perspective distortion. We chose to cut 200-μm sections, since these could be reproducibly processed for microscopy, and provide a level of anatomical detail that was suitable for comparison to the MRI contrasts. Images were restacked without any registration steps. In parallel, all sections were collected for staining (Table 2). Hematoxylin and eosin (H&E) sampling revealed no evidence of neurological or neuropathological disease, which corroborated the clinical diagnosis of nondemented control. Staining procedures were executed using standardized procedures (*59*). To minimize the overall influence of staining variations, sections were randomly assigned to different staining sessions to ensure that the interassay variation would be distributed across the brain. A detailed description of our staining procedures and initial alignment of the sections was published earlier and can be found in (*59*).

**Table 2.** Stained sections.

**Stainings**		**No. of sections** **(15-2017)**	**No. of sections** **(12-2017)**	**Rostral and caudal** staining interval*	**Central staining** interval*
Nissl (thionin)	Neuronal and glial cell bodies	253	264	1:3	1:4/5
Silver (Bielschowsky)	Nerve fibers	236	255	1:3	1:6
H&E	Neuropathological alterations	8	8	Selected anatomical levels^†^
Parvalbumin	(Inter)neurons	206	222		1:3	1:6/7
Calretinin	(Inter)neurons	45	42	-	1:6/7
Calbindin	(Inter)neurons	45	41	-	1:6/7
**Total**		793	834		

Two sections were discarded because of tissue damage in each specimen.

*Sections were defined as rostral to the corpus callosum, central (containing the corpus callosum), or caudal to the corpus callosum. Central sections were additionally sampled with calretinin and calbindin to facilitate the identification of diencephalic and metencephalic nuclei.

†Hematoxylin and eosin (H&E) staining was performed on sections containing the frontal cortex, cingulum, caudate nucleus, substantia nigra, red nucleus, hippocampus, occipital cortex, insular cortex, basal ganglia (globus pallidus and putamen), temporal cortex, subthalamic nucleus, parietal cortex, locus coeruleus, basalis pontis, cerebellar cortex, dentate nucleus, and the olivary nucleus for neuropathological assessments.

In each specimen, consecutive sections located rostral and caudal to the corpus callosum were processed for Nissl (thionin, to label glial and neuronal cell bodies), silver staining (Bielschowsky for labeling nerve fibers), and parvalbumin staining (immunolabeling of interneurons). We would like to note that Luxol fast blue staining did not provide satisfactory results in 200-μm sections for the visualization of white matter tracts. We therefore labeled nerve fibers using silver staining. In sections containing the corpus callosum, sampling intervals were increased to provide additional calretinin and calbindin labeling in both the cortex and subcortex. Calretinin and calbindin immunolabeling provide additional contrast, e.g., to facilitate the identification of individual thalamic nuclei (Fig. 1) and labeling of distinct interneuron populations in the neocortex. Intervals varied slightly (one to two sections) because of sections used for H&E staining for neuropathological diagnosis and two damaged sections. H&E sections and damaged sections were omitted from the reconstructions. After digital acquisition of the microscopy sections at 1200 pixels per inch, providing 21-μm in-plane resolution using an EPSON Perfection V700 photo (dual lens system) scanner, images were coregistered to the corresponding blockface images, which were outlined semiautomatically. We did not scan at higher resolution since our photo scanner did not allow the subsequent conversion of the large resulting files into a .tiff format. The blockface imaging volume was selected as the common space of reference as it provided sufficiently high resolution in between that of the microscopy and MRI while being free of imaging artifacts and distortions. Specimen no. 15-2017 showed a freezing artifact, which did not have any major effects on blockface imaging, although it was more prominent in the microscopy sections.

To optimize tissue contrast for Nissl-stained sections, images were acquired using a polarization filter. Color information was discarded from the images in the reconstructions to reduce data size without notable loss of information since the color did not carry any meaningful anatomical information. Individual color channels red-green-blue (RGB) contributed equally to the grayscale image. Original color information is available through the original .tiff images (Table 1).

**Fig. 4.**
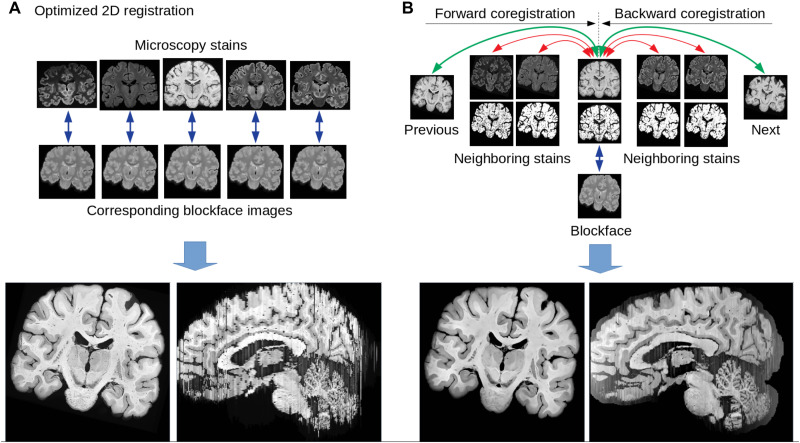
Multistaining 2D-3D coregistration. By acquiring consecutive blockface images, we reduce the registration problem to 2D-2D coregistration, which can provide good macroscopic alignment after parameter optimization (**A**). Coregistration across multiple stainings requires simultaneous alignment of each section to its neighbors and the blockface image (**B**). Using a forward-backward approach, we obtain smooth 3D reconstructions within three iterations of the registration procedure. Note that for each individual section, a separate blockface image was available, and neighboring stainings were only added to regularize the coregistration across slices and stainings. The registration pipeline code is publicly available (see Table 1).

Restacked and background-masked blockface images defined the common space, as they provided both a high-resolution 3D anatomical map and correspondence to the individual microscopy slices. Coregistration was performed using a forward-backward approach across slices, aligning individual stained contrasts not only to the blockface but also to previously coregistered neighboring slices across microscopy contrasts and feature maps outlining the contours of the brain and the position of blood vessels (Fig. 4). Alignment was performed with the ANTs SyN algorithm (*61*) and the Nighres toolbox (*62*). Coaligned microscopy images were interpolated with a nonlocal means approach (*63*) to infer contrast between stainings from combined microscopy and blockface imaging. MR images were also coaligned to the blockface in 3D with ANTs and skull-stripped using the blockface mask (Fig. 4).

Although simple linear registration of the blockface and MR images gave reasonable results, a more careful nonlinear alignment was needed to match the MRI and blockface contrasts as closely as possible. We performed a series of three coregistrations with ANTs using decreasing amounts of regularization to obtain the best results. Direct nonlinear registration with low regularization was not successful since differences resulting from some artifacts in the MRI and heterogeneity of the ventricles in the blockface images were not adequately handled by the mutual information–based coregistration. The appearance of the ventricles in the blockface images was variable because of the uneven filling with Tissue-Tek during the freezing procedure. In addition, the ventricles are subject to deformation during the mounting of the sections on glass slides for microscopy. This could be partially resolved by tailoring of the segmentation procedure to explicitly model this artifact.

Coregistering the microscopy sections to the corresponding blockface images was a challenging 2D-to-2D problem, for which we had to optimize nonlinear registration parameters (running the SyN algorithm of ANTs in a coarse-to-fine fashion over 6 scales, as defined by a scaling factor of 64 in the Nighres interface). Even with optimized 2D coregistration, the smoothness of the 3D reconstruction was still limited (Fig. 4A). To overcome this issue, we extended the registration scheme to include the alignment of a stained microscopy section not only with the corresponding blockface image but also with the two nearest coregistered neighboring slides (of any type of staining) and the nearest coregistered slide of the same staining (Fig. 4B). Because this relationship defined a Markov chain–like dependency, we could use a forward-backward approach: From the most rostral slide, we went forward to the occipital pole, including previously coregistered stains within a sliding window of three slices as they are computed and then ran the coregistration again backward from occipital to frontal pole, including only sections previously registered in the backward step. Sliding window approaches can introduce a banana effect, which is a general bending unrelated to the underlying 3D anatomy, which is avoided here by systematically including the blockface data as an anchor for the registration. The forward-backward registration step was run three consecutive times, each time using the best coregistration result in terms of mutual information as a new starting point. In addition to the staining and blockface intensities, the coregistration includes boundary and vessel maps derived from the background of the images, so that fine details such as the borders of the brain and blood vessels were better aligned. All sources of information were weighted to have equal contribution: 1 for the current slide, ^1^/_2_ for the neighboring slide of the same staining and corresponding boundary map, and ^1^/_4_ for the two nearest neighboring slides of any staining and their boundary maps (see Fig. 4B and complete coregistration script included with the data release, Table 1).

**Fig. 5.**
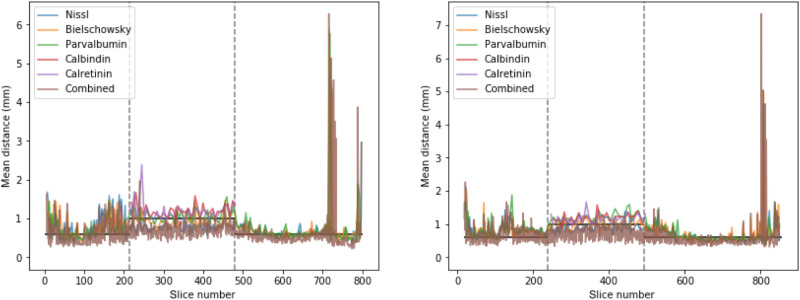
Evaluation of microscopy slice registration in both subjects: Interslice distance between boundaries of the registered microscopy images, per marker and across markers. Vertical dashed lines indicate the transitions between sampling strategies (see Table 1). Horizontal lines indicate the corresponding average interslice distance within markers. The >2-mm distances (high peaks) in the occipital pole (high slice numbers) reflect parts of the cerebellar cortex that were missing.

Coregistration of multimodal data with extensive amounts of nonlinear in-slice distortions is inherently challenging, and small registration errors may remain despite our efforts. With the data provided, fine-tuning of the coregistration in problematic cases can be done, for instance, using manual or semiautomated methods to define boundaries of interest to further optimize (*64*). To assess the quality of the coregistration, we estimated the average distance between brain boundaries and vessels measured in the microscopy images. The background separation in the blockface images was more challenging, and fine details of sulcal patterns or vasculature were often lost. We therefore omitted these in registration and evaluation results. The distances represent the average distances between consecutive slices across or within markers and show that slice-to-slice distances are on the order of the distance between them (Fig. 5). This analysis demonstrates high general accuracy and consistency of the registration. Remaining discrepancies may be further reduced in specific regions of interest by matching manual delineations or landmarks. In addition, many analyses benefit from extracting features in the original high-resolution 2D sections, rather than the 3D reconstructions, and using the coregistration transformations to combine these features into homogeneous 3D maps (e.g., Fig. 3).

In the reconstructed 3D microscopy of specimen no. 15-2017, staining-to-staining intensity differences are still visible in axial and sagittal views. In this work, rather than introduce nonlinear distortions of the staining intensity by inhomogeneity correction or nonlinear intensity matching, we resorted to linearly matching the intensity across consecutive slices of the same contrast. As a result, a small intensity drift from frontal to occipital slices can be seen in some of the contrasts, but the original dynamic range of each staining is preserved. Despite these intensity variations, the 3D reconstructions of microscopy could be used successfully to label subcortical structures with the MASSP algorithm. The automated parcellation of whole-brain structures and cortical surfaces was performed on blockface images after retuning intensity priors to match the appearance of the blockface data. Subcortical parcellations were obtained from automatized contrast mapping across the MASSP atlas and the five microscopy contrasts as well as the blockface image, following the approach described previously for parcellation on the data of the Human Connectome Project data (*48*).

## Notes

View/request a protocol for this paper from *Bio-protocol*.
