# Supplementary material for: A unified 3D map of microscopic architecture and MRI of the human brain
Source: Sci Adv. 2022 Apr 27;8(17):eabj7892. doi: 10.1126/sciadv.abj7892 (PMC9045605; doi:10.1126/sciadv.abj7892)
Supplement: Supplementary file 1 — Legend for movie S1 [file sciadv.abj7892_sm.pdf]

Science Advances
AAAS

Supplementary Materials for

A unified 3D map of microscopic architecture and MRI of the human brain

Anneke Alkemade*, Pierre-Louis Bazin, Rawien Balesar, Kerrin Pine, Evgeniya Kirilina, Harald E. Möller, Robert Trampel, Johan M. Kros, Max C. Keuken, Ronald L. A. W. Bleys, Dick F. Swaab, Andreas Herrler, Nikolaus Weiskopf, Birte U. Forstmann*

*Corresponding author. Email: jmalkemade@gmail.com (A.A.); buforstmann@gmail.com (B.U.F.)

Published 27 April 2022, *Sci. Adv.* **8**, eabj7892 (2022)
DOI: 10.1126/sciadv.abj7892

The PDF file includes:

Legend for movie S1

Other Supplementary Material for this manuscript includes the following:

Movie S1

Supplementary Movie:

Video 1: Illustration of the #15-2017 dataset. Video showing the multimodal reconstructions of the human brain, including quantitative MRI, blockface imaging and microscopy results.
